# Embryonic thermal programming combined with fractionated feeding induces early activation of hepatic lipid storage pathways in mule ducks^☆^^☆☆^

**DOI:** 10.1016/j.psj.2026.107075

**Published:** 2026-05-07

**Authors:** Laura-Lou Zwick, Joséphine Huot, Cécile Heraud, Marie Lasserre, Anne Surget, Karine Gontier, Jérôme Roy, Stéphane Panserat, Marianne Houssier

**Affiliations:** Université de Pau et des Pays de L’Adour, INRAE, NUMEA, Mont de Marsan, 40000, France

**Keywords:** Thermal programming, Fractionated feeding, Mule duck, Liver, Lipid synthesis

## Abstract

Recent studies have shown that fractionated feeding can promote the development of hyperphagic behavior in mule ducks. In parallel, embryonic thermal programming (TM) has been demonstrated to enhance hepatic energy storage under force-feeding conditions. In this exploratory study, we evaluated the combined effects of fractionated feeding and embryonic TM on short-term hepatic lipid accumulation in ducks. Two groups were compared: a CONTROL group (37.6°C during all incubation period) and a TM group (39.3°C for 16 hours per day from embryonic days 11 to 21). TM increased the embryonic relative expression of stress-response genes (*HSP90, HSP10, HSF5*) compared to controls. At 7 weeks of age, the animals underwent a 5-day acclimation period during which they had access to feed for only 1 hour per day. After this acclimation phase, ducks were offered three voluntary 1-hour meals, spaced 12 hours apart, with a commercial growth diet. Liver weight, hepatic energy composition (glycogen and lipids), and expression of genes involved in hepatic metabolism were assessed before the first meal, 6 hours after each meal, and 24 hours after the last meal. Feed intake remained high during the first two meals but decreased during the third. Despite this, liver weight and energy content increased up to 6 hours after the third meal, peaking in total lipids and oleate proportion, accompanied by upregulation of lipogenic genes expressions (*ACC, ACLY, FASN, SCD1, ELOVL6*) and downregulation of oxidative genes expressions (*ACOX1, CPT1a, ACSL1, ACAD11*). Early activation of lipogenic gene expression in the TM group led to higher liver weight and lipid content after the first meal. However, this reflected a temporal shift in lipid synthesis rather than increased fattening, as no differences were observed at the end of the protocol. Overall, embryonic thermal programming modulates the timing of hepatic lipid metabolism in ducks under voluntary fractionated feeding. This exploratory approach requires further optimization, particularly to prolong the transient hyperphagia observed. Nevertheless, it provides a first step toward understanding how nutritional strategies combined with embryonic programming may influence hepatic lipid deposition.

## Introduction

Embryonic programming refers to the set of lasting changes in an individual's metabolism or phenotype induced by environmental stimuli applied during critical periods of embryonic development. In birds, several types of programming have been explored, including light programming (Zhang et al., 2014; Drozdová et al., 2021), maternal nutrition (Ciacciariello and Tyler, 2013; Lesuisse et al., 2017), *in ovo* nutrition (Kalantar et al., 2019), and thermal programming (Loyau et al., 2013; Al Amaz and Mishra, 2024), with the aim of optimizing various zootechnical parameters such as muscle growth, meat production, or stress resistance. Embryonic thermal programming involves temporarily altering the incubation temperature of eggs (or thermal manipulation, **TM**) to induce long-lasting physiological and metabolic effects. Previous studies have shown that embryonic TM can improve thermoregulation in chickens, an effect that is thought to result from long-term molecular reprogramming (Loyau et al., 2013; Piestun et al., 2015; Al Amaz and Mishra, 2024). In particular, TM has been shown to upregulate heat shock protein (HSP)-encoding genes as well as genes involved in epigenetic regulation, supporting the existence of persistent transcriptional and epigenetic adaptations to thermal stress (Al-Zhgoul et al., 2013; Loyau et al., 2016; Vinoth et al., 2018). However, evidence in avian species indicates that embryonic TM also modulates key metabolic pathways, particularly those involved in energy and lipid metabolism. In chickens, increased incubation temperature has been shown to upregulate the expression of hepatic lipogenesis-related genes (Li et al., 2024; Amaz et al., 2024), potentially leading to increased hepatic triglyceride (**TG**) concentrations (Li et al., 2024). Similar metabolic alterations have been reported in mule ducks, where embryonic TM has been associated with increased expression of genes involved in lipid synthesis (Andrieux et al., 2023b, 2025). This capacity of embryonic TM to enhance hepatic lipid storage metabolism has been extensively investigated in mule ducks in the context of improving *foie gras* production, highlighting its potential application in the optimization of hepatic fattening processes. In force-fed ducks, it increases the liver's lipid storage capacity, leading to greater liver weight, elevated total lipid content, and overexpression of genes involved in lipogenesis (Massimino et al., 2019, 2021; Andrieux et al., 2023b).

More recently, a pioneer study conducted outside the context of force-feeding revealed that TM could also modulate liver cell size, alter lipid composition and influence metabolic gene expression following a single voluntary meal (Andrieux et al., 2023a). These data open up interesting perspectives in a context where the practice of force-feeding is increasingly criticized for animal welfare reasons, notably due to confinement and forced ingestion imposed on the animals (Council of the European Union, 1998).

The development of alternative protocols aimed at inducing voluntary hyperphagia and hepatic lipid accumulation without force-feeding therefore appears essential. In geese, nutritional strategies based on prolonged food restriction followed by *ad libitum* access have shown promising results (Guy et al., 2013; Fernandez et al., 2016; Knudsen et al., 2025). Nevertheless, these protocols prove ineffective in ducks due to their ability to rapidly regulate food intake after restriction (Knudsen et al., 2017). Interestingly, a recent study demonstrated that fractionated feeding in ducks, consisting of one-hour meals, could induce hyperphagia after an acclimation phase. When these one-hour meals were separated by 12 hours of fasting, hepatic lipid accumulation was observed after just two meals (Zwick et al., 2026). Despite the need to mobilize hepatic glycogen reserves to reveal the increase in lipid levels, and although these levels remained much lower than those observed in force-fed animals (8% vs. 60% after 21 forced meals)(Bonnefont et al., 2019), this protocol clearly demonstrated that voluntary feeding is sufficient to initiate hepatic lipid storage.

The present study investigates whether fractionated feeding, in combination with embryonic TM, can enhance hepatic lipid accumulation in ducks. The investigation focuses on two complementary axes: (i) a nutritional axis, assessing whether the addition of a third meal spaced 12 hours apart after an acclimation phase, allows the maintenance of hyperphagia and thereby increases hepatic lipid deposition; and (ii) a programming axis, using embryonic TM to induce long-term metabolic adaptations favoring hepatic lipid storage. Our hypothesis is that embryonic TM could induce lasting changes in the regulation of hepatic metabolism, promoting lipid storage through modulation of the expression of key genes involved in energy and lipid metabolism. Combined with an adapted nutritional strategy to induce voluntary hyperphagia, this approach could enhance the natural capacity of mule ducks to accumulate hepatic lipids. Here, focus is placed on short-term metabolic adaptations induced by fractionated feeding combined with embryonic TM in ducks. Without claiming to assess long-term hepatic lipid accumulation or to propose an alternative to force-feeding, it nonetheless represents an initial exploratory step aimed at evaluating the potential of this approach within this research framework.

## Materials and methods

In order to test our hypothesis, two groups of male mule ducks were compared: a control group with eggs incubated at 37.6 °C and a TM group at 39.3 °C for 16 h/day from embryonic day 11 to 21, based on previous studies (Massimino et al., 2021; Andrieux et al., 2023b). Ducks were raised until 9 weeks of age, then underwent a 5-day acclimation phase to induce hyperphagia (access to food for only 1 h/day) followed by a fattening phase consisting of three voluntarily meals spaced 12 h apart.

### Ethics approval statement

All procedures were evaluated and approved by the institutional Animal Welfare Body (SBEA). In accordance with current regulations, no specific authorization was required because the experimental design did not involve any intervention affecting animal welfare. In addition, fasting periods exceeding 12 hours are widely used in the poultry sector and have been reported not to induce weight loss or other signs of distress, thereby supporting the derogation. All experimental procedures were conducted in accordance with French national guidelines for animal care in research, at the certified Experimental Station for Waterfowl Breeding (AVIPOLE, INRAE, Artiguères, France; accreditation number B40-037-1).

### Animals

A total of 304 H85 mule duck eggs from Grimaud Frères Selection (Roussay, France) were collected on the same day and stored at 15°C for four days, to allow recovery from transport and to synchronize embryonic development, enabling sampling at the desired developmental stages without compromising survival or growth (Bagliacca et al., 2005; Onbaşılar et al., 2007). All eggs were incubated under standard conditions (37.6°C, 62.3% RH) in automated incubators with ventilation and 90° egg rotation every three hours. At embryonic day 10 (E10), candling was performed to calculate fertility rate as the percentage of fertilized eggs relative to the total eggs received. Subsequently, 130 and 129 viable eggs were randomly assigned to a CONTROL group, remaining in the original incubator, or a TM group, transferred to a separate incubator from E11 to E21. In the TM incubator, temperature was raised to 39.3°C for 16 h/day (from 4:00 p.m. to 8:00 a.m.), with humidity maintained at approximately 63.6% to prevent dehydration, following previous protocols (Massimino et al., 2021; Andrieux et al., 2023b). Incubation continued for 28 days with automatic 90° rotation every three hours, and temperature and humidity were continuously monitored every 15 min with independent sensors. At E21 (9:00 a.m., 1 h after the thermal stimulus), 16 CONTROL and 19 TM eggs were removed from the incubator (to obtain a final sample size of n = 10 viable embryos per group) for embryonic liver collection for qPCR analysis. These embryos were excluded from the calculation of embryonic mortality (LEM) and hatchability rates. This time point was selected based on the results of a previous study (Andrieux et al., 2025). A second candling at E28 was performed before transferring remaining eggs to a single hatcher (BRETAGNE model, 2,520-egg capacity, 37.3°C, 80% RH). LEM was calculated as the sum of dead embryos at E28 plus unhatched eggs per group relative to total eggs prior to E28, and hatchability as the proportion of hatched eggs relative to total fertilized eggs, including those counted before E28.

At hatching, ducklings were sexed, identified (ringed) and vaccinated with Palmivax (Boehringer Ingelheim Animal Health France, Lyon, Auvergne-Rhône-Alpes, France). The 173 hatched ducklings (107 males, 66 females) were distributed into 10 boxes, five per group (CONTROL or TM). They were vaccinated against influenza at 10 and 28 days of age and raised under identical conditions with *ad libitum* access to a starter diet (PAG1301, SOAL Haut Mauco, France; 87.8% dry matter, 40.6% starch, 17.5% protein, 2.8% fat) until 4 weeks, the density was 0.6 m² per bird and ambient temperature was maintained between 15 °C and 25 °C. After 4 weeks, they were fed an *ad libitum* growing diet (PAG1320; 87.7% dry matter, 46.5% starch, 15% protein, 2.1% fat; Supplemental file 1; Table 1) until 8 weeks.

**Table 1:** Relative expression of selected genes in the liver of embryos at the end of the thermal manipulation (E21).

Pathway	Gene	Group		Statistics		n	
		CONTROL	TM	P value	Test	CONTROL	TM
Stress	HSP90	0.83 ± 0.07	1.16 ± 0.23	0.021	t.test Welch	7	9
	HSP10	0.88 ± 0.18	1.07 ± 0.14	0.034	t.test	7	9
	HSF5	0.85 ± 0.37	1.16 ± 0.22	0.049	t.test	7	9
	UBQLN1	0.85 ± 0.14	1.21 ± 0.18	0.002	t.test	6	7
Thyroid	DIO3	0.816 ± 0.307	1.25 ± 0.19	0.004	t.test	7	9
	ALB	0.851 ± 0.441	1.37 ± 0.18	0.010	t.test	7	8
Epigenetic	ELP3	0.784 ± 0.128	1.15 ± 0.13	< 0.001	t.test	6	9
	TYMS	0.784 ± 0.14	1.16 ± 0.126	< 0.001	Wilcoxon	5	9

Genes analyzed include stress-response genes *HSP90, HSP10, HSF5*, and *UBQLN*; thyroid function and metabolism genes *DIO3* and *ALB*; and epigenetic regulation genes *ELP3* and *TYM*S. Values are presented as mean ± standard error of the mean (SEM) for each experimental group: CONTROL and TM, with individual measurements provided when available. Statistical analyses were performed using Welch’s t-test for HSP90, Student’s t-test for *HSP10, HSF5, DIO3, ALB, FGFR2* and *ELP3*, and Wilcoxon test for *UBQLN1* and *TYMS*. Different letters indicate statistically significant differences between groups at p < 0.05. Sample sizes (n) for each group are indicated.

**Fig. 1:**
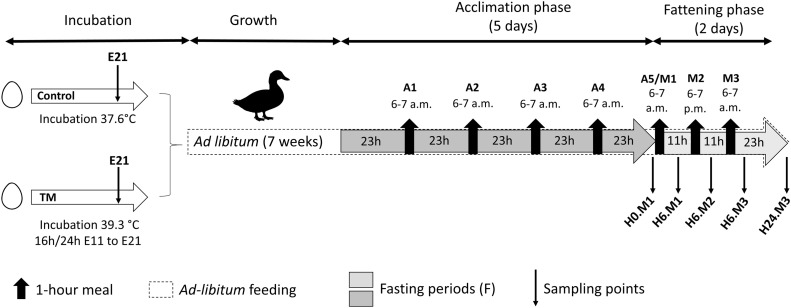
**Experimental design.** The control group (CONTROL) was incubated under standard conditions (37.6°C), while the thermally manipulated group (TM) experienced a + 1.7°C increase (39. 3°C) for 16 hours per day from embryonic day 11 (E11) to day 21 (E21). After hatching, animals (males and females) were raised under standard growth conditions. Initially fed *ad libitum*, they were then subjected to time-restricted feeding (one meal per day for 1 hour) during the acclimation phase (A1A5/M1), followed by a fattening phase with three meals spaced 12 hours apart (M1, M2 and M3). Fasting periods are shown in light and dark gray. Black arrows indicate each one-hour meals (A1, A2, A3, A4, A5-M1, M2, M3). Sampling points were defined as follows: E21 for the end of the TM period, H0.M1 (after 23 hours of fasting, before the first fattening meal; 6 a.m.), H6.M1 (6 hours after the first fattening meal; 12 a.m.) H6.M2 (6 hours after the second fattening meal; 12 p.m.), H6.M3 (6 hours after the third fattening meal; 12 a.m.), and H24.M3 (24 hours after the third fattening meal 6 a.m.).

The experimental design is shown in Fig. 1. At 7 weeks of age, all ducks underwent 5 days of food restriction (from day 52 to day 57 of rearing), with access to food for only 1 h/day distributed from 6 to 7 a.m (acclimation phase). The duration of this phase was based on a previous study from our laboratory wich demonstrated a progressive increase in consumption leading to hyperphagia by the fifth day (Zwick et al., 2026). At 8 weeks of age, the acclimation phase was followed by two additional voluntary meals spaced 12 h apart, distributed from 6 to 7 p.m. starting on day 57 of rearing, and from 6 to 7 a.m. on day 58 of rearing. These last two meals, together with the fifth meal of the acclimation phase (A5), therefore constitute the fattening phase, consisting of three meals spaced 12 hours apart (M1 to M3). During both phases, the diet provided was the growing diet (supplemental Table 1).

The mean food intake (per individual equivalent) was recorded for each meal in both phases. Liver and plasma samples were collected before the first fattening meal (H0-M1, 6 a.m.), 6 h after each fattening meal (H6-M1,12 a.m.; H6-M2, 12 p.m.; H6-M3, 12 a.m.), and 24 h after the third fattening meal (H24-M3, 6 a.m). Ducks were slaughtered by cervical bleeding by severing the blood vessels of the neck following electrical stunning (85 s, 8 pulses/s, 100 V, 20 mA), in accordance with European Council Regulation (EC) No. 1099/2009, at the Experimental Station for Waterfowl Breeding (AVIPOLE, INRAE, Artiguères, France; accreditation B40-037-1). Although both males and females were reared and included in the calculation of hatchability rates and feed intake, only data from male samples were retained for further analyses. Indeed, as TM negatively affected female hatchability, females were excluded to avoid potential bias and to ensure that the observed effects reflect programming effects rather than selection bias.

### Sampling and measurements

The sex ratio and hatchability were recorded for both males and females. Feed consumption, corresponding to pen-level standardized intake (per bird equivalent), was calculated as the difference between the amount of feed provided in the feeders and the residual feed remaining after the feeding period. This value was then divided by the number of animals with access to the feeder (from 11 to 20), to obtain the mean feed intake per animal. After slaughter, liver weights as well as body weights were determined. Liver samples were taken from the middle of the large lobe to analyze liver lipid and glycogen content, as well as gene expression levels, enzymatic activities, and were stored at −80°C. Blood samples were collected in heparinized tubes and centrifuged at 2,000 g for 10 minutes at 4°C to separate the plasma, which was then stored at −20°C until analysis.

### Plasma analyses

Plasma TG, free fatty acid (**FFA**), and glucose concentrations were determined using commercially available enzymatic assay kits according to the manufacturers’ instructions. Triglyceride levels were measured using an enzymatic assay kit (Sobioda, Montbonnot-Saint-Martin, Isère, France; product reference: W1W632-50991). Plasma glucose concentrations were quantified using a GOD-POD assay kit (Sobioda, Montbonnot-Saint-Martin, Isère, France; product reference: W1306.140), and free fatty acids were measured using the NEFA HR2 kit (Wako, Richmond, VA; product reference: W1W436-91995).

### Hepatic lipid and glycogen analysis

Liver lipid and glycogen content were analyzed using specific extraction and quantification methods. Glycogen levels were determined from 150 mg of liver tissue. Initially, free glucose was measured from homogenized samples in 1 N hydrogen chloride (**HCl**). The remaining glycogen was then hydrolyzed by heating the samples at 100°C for 2 hours and 30 minutes in HCl. Free glucose and total glucose (post-hydrolysis) were quantified using an enzymatic assay Glucose GOD-POD (Sobioda, Montbonnot-Saint-Martin, Isère, France; product reference: W1306.140). Hepatic glycogen content was calculated by subtracting the free glucose fraction from the total glucose and normalizing it to 150 mg of liver tissue.

For lipid analysis, hepatic TG were first extracted from 100 mg of liver tissue, homogenized in 1 ml of a 5% Igepal detergent solution, and subjected to two heating cycles at 90°C for 5 minutes each. The TG content was then assessed using an enzymatic assay for Triglycerides (Sobioda, Montbonnot-Saint-Martin, Isère, France; product reference: W1W632-50991) following the manufacturer's guidelines.

Total lipids were extracted separately from 5 g of liver samples using an Ultra-Turrax homogenizer in a dichloromethane/methanol mixture (apolar/polar, 2:1 v/v) containing 0.1% butylated hydroxytoluene (**BHT**) (Sigma-Aldrich, St. Louis, MO; product reference: B1378) to prevent lipid oxidation. The lipids were then extracted according to the Folch method (Folch et al., 1957; Cequier-Sánchez et al., 2008). After determining the total lipid percentage by weighing, the extracted lipids were suspended in dichloromethane/methanol (0.1% BHT m/v) mixture. Triglyceride samples were first saponified under heat with 0.5 M methanolic potassium hydroxide. The resulting free fatty acids were then methylated by heating with 14% (v/v) boron trifluoride (BF₃) to form fatty acid methyl esters (**FAMEs**). FAMEs were extracted with hexane, and the organic phase was washed with water to remove residual reagents. Finally, the hexane extract was dried over anhydrous sodium sulfate (Na₂SO₄) prior to further analysis. Fatty acids were separated by gas chromatography coupled to a Flame Ionization Detector (**GC-FID**, Varian 3900) with an injector in Split/Splitless mode, using a capillary column (DB-wax × 30 m × 0.25 mm). The operating parameters were: column heating temperature of 100°C for injection followed by heating ramp of 180°C (8°C/min) for 10 min and a final heating ramp of 220°C (4°C/min) for 25 min. The injector and detector temperatures were kept at 260°C and 250°C, respectively. Gas flows were 1 mL/min for carrier gas (H_2_), 25 mL/min for make-up gas (He), and for FID 30.0 and 300.0 mL/min for H_2_ gas and air, respectively. Injections of 1 μL were performed in split mode at a 1/40 ratio. Fatty acid identification and proportion were performed using Star Chromatography workstation 6.0 (Varian) and adapted standard mixtures.

### Gene expressions measurements

RNA was extracted from embryonic and adult liver samples using TRIzol (Thermo Fisher Scientific, Waltham, MA; product reference: #12034977) following the manufacturer’s protocol. RNA quality was checked on a 1% agarose gel and concentration measured at 260 nm with a Biotek EPOCH 2 reader. All samples were then normalized to 500 ng/µl.

Reverse transcription (**RT**) was performed in duplicate using 1 µg RNA in 10 µl RNase-free water. The reaction included Random Primers (Promega, Madison, WI; product reference: C1181, 0.2 µg/ml final), a PCR nucleotides mix (Promega, Madison, WI; product reference: C1145, 0.004 mM final), and exogenous luciferase RNA (1 pg; 0.1 pg/µl final). RNA was first processed in a CFX384 thermal cycler (BioRad, Hercules, CA) with an initial 65 °C step followed by cooling at 4 °C. Reverse transcription of 1 µg total RNA (0.05 µg/µl final) was carried out using SuperScript III (Invitrogen, Waltham, MA, product reference; 18080044, 5 U/µl final) and RNaseOUT (Invitrogen; 10777019, 2 U/µl final), following the program: 25°C for 5 min, 55°C for 1 h, and 70°C for 15 min. To ensure reaction specificity and the absence of contamination, negative controls were included for each group: a no-RT control (without RNA) and a no-DNA control (without SuperScript).

Relative gene expression was quantified by real-time qPCR using Perfecta SYBR Green FastMix (VWR, Radnor, PA; product reference: 733-1379). Each 6-µl reaction contained 2 µl of 1:80-diluted cDNA, 3 µl SYBR Green mix, and gene-specific primers at 400 nM final (Supplemental file 1; Table 2). The qPCR reactions were performed on a CFX384 system (BioRad, Hercules, CA) using a standard amplification program (95 °C pre-incubation, followed by 45 cycles at 95 °C, 60 °C, and 72 °C). Primer efficiency was validated through serial dilutions, retaining only primers with efficiencies >85%. Melting-curve analysis confirmed single-product amplification, and negative DNA and RT controls verified the absence of contamination. cDNA of reference genes, target genes, and luciferase (exogenous RNA control) was amplified for each RT replicate (RT1 and RT2). Ct values were averaged across technical replicates, and samples showing a Ct difference greater than 1 between RT1 and RT2 were excluded from the analysis to ensure accurate and consistent gene expression measurements.

**Table 2:** Incubation and hatching outcomes for CONTROL and TM groups.

	Fertility rate (%)	LEM (%)	Hatchability (%)	male proportion (%)	n male	n female
Control	84.41	15.92^b^	84.07^a^	55.78^b^	53	42
TM		29.72^a^	70.2^b^	69.23^a^	54	24
p value	-	< 0.001	< 0.001	0,017	-	-
test	-	χ² test	χ² test	χ² test	-	-

Fertility rate and late egg mortality (LEM, %), hatchability (%), proportion of males (%), and the number of males (n male) and females (n female) hatched are presented. Statistical analyses were performed as follows: LEM, hatchability, and proportion of males were analyzed using χ² tests with theoretical probabilities based on the CONTROL group. Different letters indicate statistically significant differences between groups (p < 0.05). Sample sizes (n) for each group are indicated*.*

The relative expression of the genes of interest was calculated using the Livak and Schmittgen (2001) method, based on the obtained Ct values. For each sample type, the most stable reference genes were selected. Reference genes were selected independently for embryonic and adult liver samples based on their expression stability across experimental groups, assessed by both minimal variation in mean expression between conditions and the lowest standard deviations within each group. This approach ensured that only genes exhibiting consistent expression under the specific physiological contexts were retained for normalization. In addition, exogenous luciferase was included as an external control to account for potential technical variability and, notably, because developmental (embryonic stages) and metabolic (fasting vs. refeeding) conditions may still induce variability even in commonly used stable housekeeping genes. For embryonic liver samples, the normalization was performed using the geometric mean of the Cts of luciferase and *ActinB* as the reference genes. For adult liver samples, the normalization was done using the geometric mean of the Cts of *EF1* and luciferase as the reference genes. The relative expression was calculated as follows:

Relative expression = (RQ sample) / (RQ reference), where the RQ sample = 2^ΔCt sample and RQ reference = 2^ΔCt reference. ΔCt sample is the difference between the average Cts of all samples and the Ct of the sample for target gene, and ΔCt reference is the difference between the average Cts of all samples and the Ct of one sample for reference genes. This method allowed for the normalization of gene expression in relation to stable reference genes, ensuring accurate comparisons across samples.

### Statistical analysis

Statistical analyses were conducted using R software version 3.6.2 with the RStudio interface. A chi-square test was first performed to analyze the LEM, hatchability and sex ratio measurements. Gene expression data in embryonic liver samples, the application conditions were first validated using the Shapiro-Wilk test for normality and the Levene test for homoscedasticity. If both normality and homoscedasticity were respected (p-value > 0.05), a t-test was applied. If homoscedasticity was not met, Welch’s t-test was used, and if normality was violated, a Wilcoxon test was applied.

For adult liver gene expression data, as well as liver weight, hepatic energy composition (glycogen, triglycerides, and total lipids), fatty acid profiles, plasma concentrations (glucose, triglycerides, and free fatty acids), hepatic gene expressions, and enzymatic activities, the analysis followed a two-way ANOVA procedure. Normality was assessed using the Shapiro-Wilk test, and homoscedasticity was verified with Levene’s test. If both assumptions were met, a two-way ANOVA followed by Tukey’s post-hoc test was applied. If at least one assumption was not satisfied, a log transformation was applied before performing the two-way ANOVA, followed by Tukey’s post-hoc test. If the log transformation was insufficient to meet the assumptions, the subsequent analysis depended on which assumption remained violated: when only homoscedasticity was not fulfilled, a Generalized Least Squares (**GLS**) model was applied, either on log-transformed or raw data depending on which best met model assumptions; when both assumptions remained violated or when the sample size was too small (n < 6), a Scheirer-Ray test was used, followed by Dunn’s post-hoc test. The two-way ANOVA, Scheirer-Ray test and GLS analyzed the effects of nutritional condition (**NC**), which included H0-M1, H6-M1, H6-M2, H6-M3, and H24-M3, as well as the effect of thermal manipulation (CONTROL vs. TM). The F value, Mean Sq, and degrees of freedom (**Df**) were calculated to assess significance and variance within each group. Results were considered statistically significant if p-value < 0.05. The experimental conditions, in which animals feed voluntarily and therefore show inter-individual differences in intake, may lead to substantial variability in responses, particularly in postprandial conditions. This heterogeneity requires statistical tests that account for unequal variances between groups. The use of different statistical tests ensured that the most appropriate statistical framework was applied to each dataset according to its distributional properties and sample size.

## Results

### Embryonic relative expression of key genes at the end of the thermal manipulation

To confirm that the TM group properly perceived the temperature increase, the relative expression (Table 1) of genes known to be responsive to temperature change was analyzed in the livers of embryos collected at the end of the thermal manipulation (E21). The expression of four target genes involved in the heat stress pathway (*HSP90, HSP10, HSF5* and *UBQLN1*) as well as genes involved in thyroid function (*DIO3* and *ALB*) and epigenetic marks (*ELP3* and *TYMS*) increased in the TM group compared to control.

### Impact of thermal manipulation on embryonic parameters and hatchability

Measures of late embryonic mortality (LEM), hatchability and sex ratio (proportion of males) are presented in Table 2. First, a significant increase in LEM was measured in the TM group (29.72%) compared to the CONTROL group (15.92%) (p < 0.001). Regarding hatchability, the CONTROL group showed a higher rate (84.07%) than the TM group (70.2%) (p < 0.001). In contrast, the sex ratio revealed a significantly higher proportion of males in the TM group (69.23%) compared to the CONTROL group (55.78%) (p = 0.017).

### Feed intake during the acclimation phase and the fattening phase in the CONTROL and TM groups

**Fig. 2:**
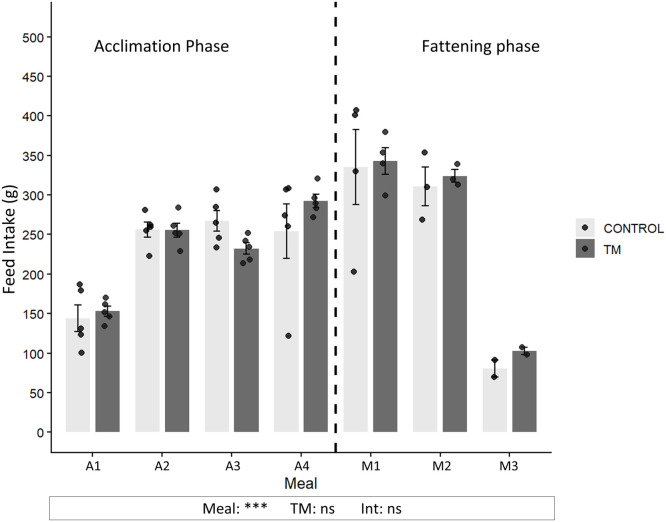
**Feed consumption, corresponding to pen-level standardized intake (g per bird equivalent) in CONTROL and TM groups.** Bars represent mean ± sd. (standard deviation). Individual data points are shown to illustrate within group variability and correspond to the total food consumed per pen divided by the number of animals with access to the feeder (n=8-2). Meals A1-A4 correspond to the acclimation phase (meals spaced 23 h apart), A5-M1 represents the transition meal, and M2-M3 correspond to the fattening phase (meals spaced 12 h apart). A dashed line separates these two phases. Statistical analysis was performed using the non-parametric Scheirer-Ray-Hare test (n < 6), assessing the main effects of Meal (A1, A2, A3, A4, A5-M1, M2, M3), TM (treatment condition), and their interaction (Meal x TM). Significance levels are indicated as follows: ***p < 0.001, **p < 0.01, *p < 0.05, and ns (not significant) p ≥ 0.05, n = 2 to 5.

***Meal Effect.*** Pen-level standardized intake (per bird equivalent) was measured for each meal during the acclimation phase (spaced 23 hours apart) and the fattening phase (spaced 12 hours apart) (Fig. 2). Feed intake was significantly influenced by the “Meal” effect, corresponding to the different meals offered to the animals (A1, A2, A3, A4, A5-M1, M2, M3; p < 0.001). A progressive increase in consumption was measured between the first (A1) and the fifth (A5-M1) meal during the acclimation phase (from 144 ± 37.3 to 335 ± 94.4 g (2.33-fold increase) for CONTROL group and from 153 ± 14.1 to 343 ± 33.6 g (2.24-fold increase) for TM group; p < 0.001). During the fattening phase (with meals spaced 12 hours apart), the average feed intake was maintained above 300 g for both groups during the first two fattening meals (A5-M1 and M2) but markedly decreased at meal M3. For the CONTROL group, intake dropped from 311 ± 42.5 g to 80.4 ± 15.2 g, representing a reduction of approximately 74%, while for the TM group, intake decreased from 343 ± 33.6 g to 103 ± 6.6 g, corresponding to a reduction of about 70% (p < 0.001).

***Thermal Manipulation condition Effect.*** The mean feed intake over all meals spaced 23 hours apart (from A1 to A5-M1) was 255 g for the CONTROL group and 255.6 g for the TM group. For meals spaced 12 hours apart (from A5-M1 to M3), the average intake was 242.1 g for the CONTROL group and 256.7 g for the TM group. No significant difference in feed intake was observed between the CONTROL and TM.

### Changes in plasma parameters during the fattening phase in the CONTROL and TM groups

**Fig. 3:**
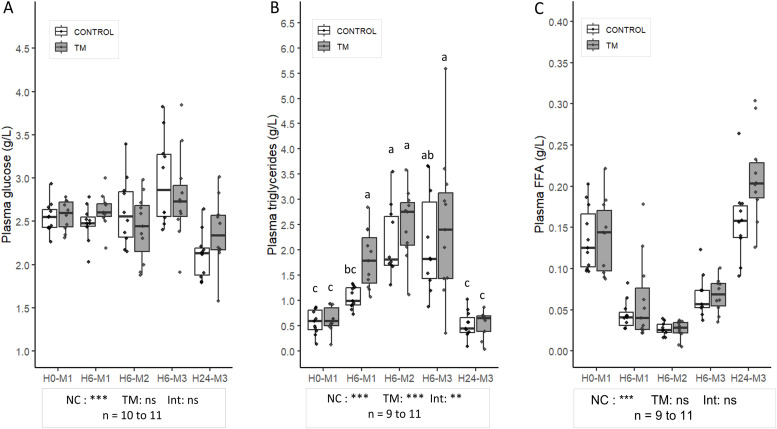
**Plasma glucose (A), triglyceride (B), and free fatty acid levels (C) in CONTROL and TM groups.** The boxplots represent the distribution of each variable, showing the median, interquartile range (IQR), and individual data points to illustrate within-group variability. A generalized least squares (GLS) model was applied to analyze plasma glucose and triglyceride (**TG**) levels, accounting for variance heterogeneity between groups. For plasma free fatty acids (**FFA**), data were analyzed using the non-parametric Scheirer-Ray-Hare test, followed by Dunn’s post-hoc test when appropriate. Significance levels are indicated as follows: ***p < 0.001, **p < 0.01, *p < 0.05, and ns (not significant) p ≥ 0.05. NC: effect of nutritional condition; TM: effect of thermal manipulation; Int: interaction between the two factors. Different letters indicate statistically significant differences between groups at p < 0.05. Sample sizes (n) for each group are indicated.

***Nutritional Condition Effect*.** The plasma parameters analyses are presented in Fig. 3. A decrease in plasma glucose was measured for both groups at H24-M3, 24 hours after the third fattening meal (2.11 ± 0.26 g/L for CONTROL and 2.37 ± 0.41 g/L for TM) compared to H0-M1 (2.55 ± 0.18 g/L for CONTROL and 2.57 ± 0.17 for MT; p = 0.007) and H6-M3 (2.97 ± 0.52 g/L for CONTROL and 2.78 ± 0.52 g/L for MT; p < 0.001). Six hours after each fattening meal an increase in TG levels was observed compared to H0 in TM group (maximum reach at H6-M2 with 2.52 ± 0.69 g/L) whereas in the control group, this increase was only observed after the second fattening meal (H6-M2) (from 0.58 ± 0.24 g/L to 2.17 ± 0.71), before returning to its initial level 24 hours after the third meal (H24-M3). Finally, plasma FFA concentrations were also affected by nutritional conditions (p < 0.001), initially decreasing from 0.136 ± 0.039 g/L (CONTROL) and 0.139 ± 0.045 g/L (MT) at H0-M1 to 0.027 ± 0.008 (CONTROL) and 0.026 ± 0.011 g/L (MT) at H6-M2 (p < 0.001), before rising again and returning to their initial level at H24-M3.

***Thermal Manipulation condition Effect.*** No significant impact of TM was observed on plasma glucose or FFA concentrations, in contrast to plasma TG levels which were higher on average for the TM group (1.65 ± 1.15 g/L) compared to the CONTROL group (1.26 ± 0.91 g/L) (p = 0.025). Additionally, an interaction with nutritional condition (NC) was observed (p = 0.012), with plasma TG levels approximately 72% higher in the TM group (1.82 ± 0.58 g/L) compared with the CONTROL group at H6-M1 (1.06 ± 0.21 g/L) (p = 0.015).

### Body weights, liver weights and energy compositions during the fattening phase in the CONTROL and TM groups

**Fig. 4:**
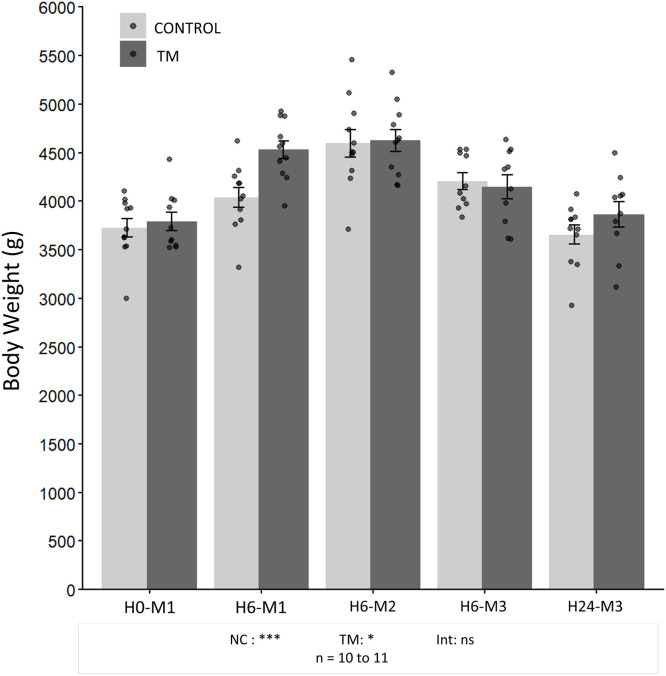
**Evolution of body weight in the CONTROL and TM groups**. Results are presented as bar plots showing mean ± SD. Individual values are displayed as points to illustrate within-group variability. A two-way ANOVA was performed, followed by Tukey’s post hoc test. Significance levels are indicated as follows: ***p < 0.001, **p < 0.01, *p < 0.05, and ns (not significant) p ≥ 0.05. NC: effect of nutritional condition; TM: effect of thermal manipulation; Int: interaction between the two factors.

**Fig. 5:**
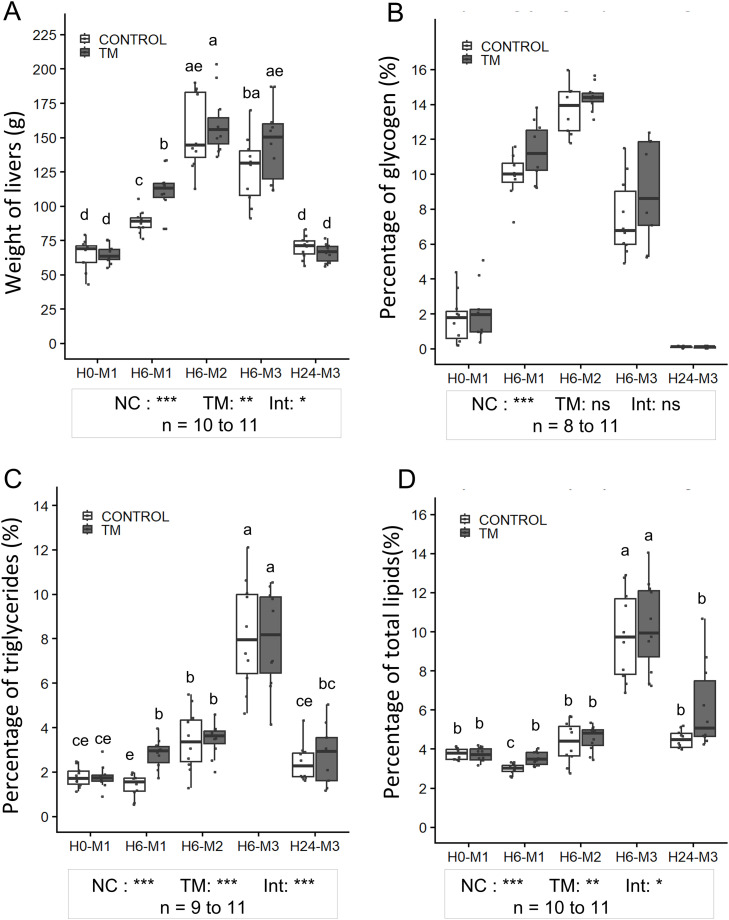
**Liver weight (A) and hepatic glycogen (B), triglyceride (C), and total lipid (D) levels in CONTROL and TM groups.** The boxplots represent the distribution of each variable, displaying the median, interquartile range (IQR), and individual data points to illustrate within-group variability. For liver weight (A), data were log-transformed before performing a two-factor ANOVA, followed by Tukey's post-hoc test. For hepatic glycogen and triglyceride percentages (B-C), generalized least squares (GLS) models were applied on the raw data, followed by Tukey's post-hoc test. For hepatic total lipid percentages (D), GLS was applied on log-transformed data, followed by Tukey's post-hoc test. Statistical significance is indicated as follows: ***p < 0.001, **p < 0.01, *p < 0.05, and ns (not significant) p ≥ 0.05. NC: effect of nutritional condition, TM: thermal manipulation, Int: interaction effect. Different letters indicate statistically significant differences between groups at p < 0.05. Sample sizes (n) for each group are indicated.

***Nutritional Condition Effect.*** Body weight, presented in Fig. 4, was significantly affected by the nutritional condition of the animals (NC) (p < 0.001). It increased from 3726 ± 315 g (H0-M1) to 4597 ± 463 g (H0-M2) in the CONTROL group (p < 0.001), and from 3793 ± 301 g (H0-M1) to 4627 ± 372 g (H0-M2) in the TM group (p < 0.001). Thereafter, it decreased, returning to levels comparable to baseline values 24 h after the third meal. As shown in Fig. 4, liver weight and the percentages of glycogen, triglycerides, and total hepatic lipids were significantly influenced by nutritional condition (NC) (p < 0.001 for all parameters). Liver weight (Fig. 5.A) and hepatic glycogen levels (Fig. 5.B) followed similar patterns, with a progressive increase between H0-M1 and H6-M2. Liver weight increased approximately 2.37-fold in the CONTROL group (from 64.81 ± 10.69 g to 153.31 ± 27.06 g) and about 2.46-fold in the TM group (from 64.9 ± 6.75 g to 159.54 ± 21.61 g) (p < 0.001). Hepatic glycogen content similarly rose from 1.71 ± 1.34% (CONTROL) and 2.10 ± 1.58% (TM) to 13.72 ± 1.38% (CONTROL) and 14.40 ± 0.75% (TM) (p < 0.001). At H6-M3, both liver weight and glycogen content began to decrease and continued to decline until H24-M3, when they returned to their initial levels, with glycogen even approaching complete depletion of stores (0.10 ± 0.05% for CONTROL and 0.085 ± 0.05% for TM).

Hepatic levels of TG (Fig. 5.C) and total lipids (Fig. 5.D) followed a different pattern, with a significant increase between H0-M1 and H6-M3 (p < 0.001). Hepatic TG increased 4.6-fold in the CONTROL group (from 1.77 ± 0.45% to 8.18 ± 2.45%) and 4.5-fold in the TM group (from 1.78 ± 0.52% to 8.00 ± 2.15%). Total lipids increased about 2.6-fold in the CONTROL group (from 3.74 ± 0.27% to 9.83 ± 2.24%) and 2.8-fold in the TM group (from 3.73 ± 0.34% to 10.28 ± 2.23%). Both parameters returned to baseline at H24-M3.

***Thermal Manipulation condition Effect:*** Regarding body weight, a significant overall effect of thermal manipulation was observed (p = 0.028), with a higher overall mean in the TM group compared to the CONTROL group (4208 ± 485 g vs. 4041 ± 484 g). No interaction effect between TM and nutritional condition was observed for this parameter (p = 0.104). The overall analysis indicated that the mean liver weight was higher in the TM group compared to the CONTROL group (p = 0.004). Furthermore, an interaction between nutritional condition and TM indicated a significant increase in liver weight in the TM group compared to the CONTROL group at H6-M1, with liver weight being 24.8% higher in the TM group (110.64 ± 16.24 g versus 88.64 ± 7.77 g respectively) (p = 0.034). Although hepatic glycogen percentages followed a trend similar to liver weight according to nutritional condition, no significant effect of TM was observed. In contrast, hepatic TG and total lipid percentages were affected (p < 0.001 and p = 0.002, respectively), and an interaction between TM and nutritional condition was measured (p = 0.027 and p = 0.019, respectively). For both parameters, increases in the TM group were observed at H6-M1 compared to control group (p < 0.001 for hepatic TG and p = 0.024 for total lipids), with hepatic TG approximately 2.0-fold higher in the TM group (2.83 ± 0.65% versus 1.39 ± 0.48%) and total lipids about 17% higher (3.52 ± 0.35% versus 3.00 ± 0.26%).

### Fatty acid composition of livers during the fattening phase in the CONTROL and TM groups

Table 3 presents the fatty acid composition of total hepatic lipids. The percentages correspond to the proportion of each fatty acid relative to the total detected fatty acids.

**Table 3:** Hepatic fatty acid composition (%) in CONTROL and TM groups.

		Nutrionnal Condtion (NC)					Anova two ways or Scheirer–Ray–Hare test			
	Group	H0-M1	H6-M1	H6-M2	H6-M3	H24-M3	NC effect	TM effect	Interaction	n
		23 h fast	6 h post meal	6 h post meal	6 h post meal	23 h fast	P value	P value	P value	
SFA Percentage of each fatty acid over the total detected fatty acids										
Sum SFA %	CONTROL	40.0 ± 0.88^c^	44.4 ± 1.52^b^	45.0 ± 3.16^ab^	41. 7 ± 1.50^c^	38.9 ± 1.03^cd^	< 0.001	0.895	< 0.001	7 to 11
	TM	37.1 ± 0.93^d^	47.4 ± 1.69^a^	46.5 ± 2.03^ab^	39.8 ± 2.22^cd^	38.9 ± 1.56^cd^				
C14:00%	CONTROL	0.166 ± 0.047	0.224 ± 0.089	0.405 ± 0.088	0.543 ± 0.031	0.149 ± 0.034	< 0.001	0.049	0.578	7 to 11
	TM	0.222 ± 0.036	0.354 ± 0.075	0.405 ± 0.071	0.549 ± 0.115	0.221 ± 0.113				
C15:00%	CONTROL	0.023 ± 0.014	0.024 ± 0.018	0.015 ± 0.014	0.007 ± 0.01	0.024 ± 0.02	< 0.001	0.004	0.838	6 to 10
	TM	0.014 ± 0.018	0.019 ± 0.014	0	0	0.006 ± 0.011				
C16:00%	CONTROL	21.0 ± 1.38	24.0 ± 1.37	29.2 ± 2.0	27.6 ± 1.68	21.7 ± 1.55	< 0.001	0.166	0.135	7 to 10
	TM	19.7 ± 1.33	29.6 ± 1.65	29.4 ± 2.14	27.7 ± 2.62	22.3 ± 1.57				
C17:00%	CONTROL	0.101 ± 0.093^ac^	0.014 ± 0.044^ab^	0.017 ± 0.026^abc^	0.013 0.02^abc^	0.0^b^	< 0.001	0.097	< 0.001	7 to 10
	TM	0.0^ab^	0.101 ± 0.073^c^	0.028 ± 0.036^abc^	0.0^b^	0.0^ab^				
C18:00%	CONTROL	18.4 ± 1.74	19.1 ± 1.77	15.2 ± 1.84	13.5 ± 0.817	16.5 ± 1.48	< 0.001	0.117	0.389	7 to 11
	TM	17.1 ± 0.876	17.0 ± 1.56	16.2 ± 1.33	11.5 ± 0.692	16.3 ± 1.65				
MUFA Percentage of each fatty acid over the total detected fatty acids										
Sum MUFA %	CONTROL	19.4 ± 3.71^ef^	17.9 ± 1.48^f^	39.4 ± 3.2^b^	46.6 ± 1.64^a^	33.7 ± 1.52^c^	< 0.001	< 0.001	< 0.001	7 to 11
	TM	23.6 ± 3.96^de^	26.3 ± 1.36^d^	35.0 ± 2.94^bc^	46.5 ± 2.47^a^	33.8 ± 7.54^c^				
C16:1%	CONTROL	0.723 ± 0.254^ef^	0.506 ± 0.168^f^	2.44 ± 0.44^b^	2.67 ± 0.33^b^	0.701 ± 0.13^ef^	< 0.001	0.004	< 0.001	7 to 11
	TM	0.855 ± 0.219^ef^	1.48 ± 0.554^cd^	1.72 ± 0.372^c^	3.38 ± 0.575^a^	1.06 ± 0.453^de^				
C18:1%	CONTROL	18.1 ± 3.25^de^	16.9 ± 1.45^e^	36.5 ± 3.10^b^	43.6 ± 1.80^a^	32.2 ± 1.54^b^	< 0.001	< 0.001	< 0.001	7 to 11
	TM	22.1 ± 3.62^cd^	24.2 ± 1.31^c^	32.7 ± 2.81^b^	42.7 ± 2.62^a^	32.1 ± 7.03^b^				
C20:1%	CONTROL	0.259 ± 0.092	0.415 ± 0.3	0.308 ± 0.13	0.439 ± 0.2	0.414 ± 0.21	0.471	0.415	0.055	7 to 10
	TM	0.357 ± 0.09	0.547 ± 0.345	0.31 ± 0.066	0.285 ± 0.11	0.355 ± 0.103				
PUFA n6 Percentage of each fatty acid over the total detected fatty acids										
Sum PUFA n6%	CONTROL	34.9 ± 2.42	33.9 ± 1.76	13.8 ± 2.42	10.3 ± 2.28	23.7 ± 1.21	< 0.001	0.089	0.419	7 to 10
	TM	32.4 ± 1.85	22.8 ± 2.32	14.9 ± 2.00	9.01 ± 1.49	22.8 ± 6.52				
C18:2 n6%	CONTROL	7.33 ± 0.741^b^	11.7 ± 1.77^a^	5.4 ± 0.87^cd^	4.13 ± 0.925^de^	5.01 ± 1.04^de^	< 0.001	0.016	< 0.001	6 to 11
	TM	6.71 ± 0.781^bc^	8.16 ± 0.939^b^	5.54 ± 0.212^cd^	3.74 ± 0.386^e^	5.44 ± 0.994^cd^				
C18:3 n6%	CONTROL	0.082 ± 0.072	0.058 ± 0.062	0.085 ± 0.092	0.069 ± 0.065	0.006 ± 0.011	0.002	0.65	0.089	7 to 11
	TM	0.059 ± 0.067	0.067 ± 0.06	0.138 ± 0.09	0	0.011 ± 0.014				
C20:2 n6%	CONTROL	0.522 ± 0.085^bc^	0.396 ± 0.101^bc^	0.375 ± 0.078^bc^	0.596 ± 0.254^ab^	0.82 ± 0.274^a^	< 0.001	0.459	0.002	7 to 11
	TM	0.84 ± 0.183^a^	0.324 ± 0.041^c^	0.408 ± 0.067^bc^	0.591 ± 0.179^ab^	0.822 ± 0.235^a^				
C20:3 n6%	CONTROL	0.823 ± 0.119	0.746 ± 0.177	0.907 ± 0.221	0.615 ± 0.25	0.824 ± 0.424	0.001	0.256	0.157	7 to 11
	TM	1.04 ± 0.187	0.676 ± 0.059	0.833 ± 0.136	0.639 ± 0.17	0.658 ± 0.192				
C20:4 n6%	CONTROL	20.2 ± 2.04	16.0 ± 1.66	5.01 ± 1.12	3.77 ± 1.14	12.8 ± 1.05	< 0.001	0.217	0.573	7 to 10
	TM	18.5 ± 2.31	9.66 ± 2.02	6.00 ± 1.14	3.07 ± 0.706	11.9 ± 4.96				
C22:4 n6%	CONTROL	2.14 ± 0.261	1.88 ± 0.323	0.802 ± 0.253	0.369 ± 0.197	1.70 ± 0.159	< 0.001	< 0.001	0.077	7 to 11
	TM	1.88 ± 0.24	1.42 ± 0.276	0.736 ± 0.18	0.32 ± 0.09	1.42 ± 0.349				
C22:5 n6%	CONTROL	3.50 ± 0.566	2.29 ± 0.439	0.717 ± 0.334	0.472 ± 0.254	2.17 ± 0.581	< 0.001	0.226	0.421	7 to 11
	TM	3.00 ± 0.586	2.04 ± 0.361	0.805 ± 0.269	0.425 ± 0.216	2.00 ± 0.666				
PUFA n3 Percentage of each fatty acid over the total detected fatty acids										
PUFA n3, %	CONTROL	3.08 ± 0.304^a^	2.38 ± 0.469^bc^	1.07 ± 0.317^e^	0.688 ± 0.239^e^	2.02 ± 0.598^bc^	< 0.001	0.013	0.006	6 to 11
	TM	2.65 ± 0.204^ab^	1.76 ± 0.468^cd^	1.13 ± 0.164^de^	0.897 ± 0.522^e^	1.79 ± 0.647^cd^				
C16:4 n3	CONTROL	0.231 ± 0.042	0.187 ± 0.062	0.095 ± 0.063	0.044 ± 0.028	0.193 ± 0.051	< 0.001	0.024	0.109	6 to 11
	TM	0.228 ± 0.053	0.12 ± 0.05	0.109 ± 0.046	0.027 ± 0.023	0.137 ± 0.075				
C18:3 n3	CONTROL	0.116 ± 0.076	0.093 ± 0.131	0.042 ± 0.063	0.024 ± 0.037	0	0.021	0.794	0.071	7 to 11
	TM	0.029 ± 0.049	0.132 ± 0.109	0.056 ± 0.07	0.008 ± 0.016	0.039 ± 0.054				
C20:5 n3	CONTROL	0.097 ± 0.055^a^	0.192 ± 0.1^a^	0.224 ± 0.143^a^	0.136 ± 0.096^a^	0.106 ± 0.032^a^	0.215	0.308	0.03	6 to 11
	TM	0.324 ± 0.196^a^	0.235 ± 0.238^a^	0.132 ± 0.062^a^	0.485 ± 0.431^a^	0.108 ± 0.062^a^				
C21:5 n3	CONTROL	0.117 ± 0.018	0.088 ± 0.039	0.046 ± 0.046	0.034 ± 0.035	0.067 ± 0.043	< 0.001	0.085	0.983	7 to 11
	TM	0.098 ± 0.045	0.062 ± 0.037	0.031 ± 0.03	0.025 ± 0.04	0.053 ± 0.045				
C22:5 n3	CONTROL	0.303 ± 0.207	0.127 ± 0.045	0.087 ± 0.025	0.078 ± 0.048	0.196 ± 0.179	< 0.001	0.554	0.209	7 to 11
	TM	0.176 ± 0.161	0.16 ± 0.079	0.125 ± 0.045	0.022 ± 0.027	0.222 ± 0.165				
C22:6 n3	CONTROL	1.93 ± 0.45^a^	1.55 ± 0.22^ab^	0.484 ± 0.189^d^	0.313 ± 0.125^d^	1.51 ± 0.332^ab^	< 0.001	0.024	< 0.001	7 to 11
	TM	1.76 ± 0.16^a^	1.04 ± 0.259^c^	0.592 ± 0.149^d^	0.284 ± 0.135^d^	1.21 ± 0.446^bc^				

Values are presented as mean ± standard deviation. Saturated fatty acids (**SFA**), monounsaturated fatty acids (**MUFA**), and polyunsaturated fatty acids (PUFA, n3 and n6) are expressed as the percentage of each fatty acid over the total detected fatty acids. Statistical analyses were performed using two-way ANOVA on raw data for sum SFA %, C22:4 n6, C22:5 n6, and C16:4 n3; ANOVA on log-transformed data for C18:2 n6; Scheirer–Ray–Hare non-parametric test on raw data for C14:0, C15:0, C16:0, C17:0, C18:0, C20:1, sum PUFA n6, C18:3 n6, C20:3 n6, C20:4 n6, C18:3 n3, C20:5 n3, C21:5 n3, and C22:5 n3 followed by Dunn’s post-hoc test; and generalized least squares (GLS) on raw data followed by Tukey’s post-hoc test for sum MUFA %, C16:1, C18:1, C20:2 n6, PUFA n3, and C22:6 n3. Effects of nutrictional condition (NC), thermal manipulation (TM), and their interaction are reported with F-values or P-values as appropriate. Different letters indicate statistically significant differences between groups at p < 0.05. Sample sizes (n) for each group are indicated. Different letters indicate statistically significant differences between groups at p < 0.05.

***Nutritional Condition Effect.*** An increase in the proportion of saturated fatty acids (**SFA**) among total fatty acids was observed between H0-M1 and H6-M1, rising from 40.0 ± 0.88% to 44.4 ± 1.52% for the CONTROL group (an increase of approximately 11%) and from 37.1 ± 0.93% to 47.4 ± 1.69% for the TM group (an increase of 27.8%) (p < 0.001). This increase was mainly explained by the marked rise in palmitic acid (C16:0), the predominant FA of this family. Subsequently, the percentage of SFA returned to its initial level within 6 hours after the third fattening meal (H6-M3).

An ensuing increase in monounsaturated fatty acids (**MUFAs**) was observed with the percentage rising significantly from 19.4 ± 3.71% before the first meal to 46.6 ± 1.64% after the third meal for the CONTROL group (increase of 2.4-fold) and from 23.6 ± 3.96% to 46.5 ± 2.47% for the TM group (increase of 2.0-fold) (p < 0.001). Although the levels subsequently declined, they remained significantly elevated 24 hours after the last meal at 33.7 ± 1.52% (CONTROL) and 33.8 ± 7.54% (TM) compared to H0-M1 (p < 0.001). This dynamic was primarily driven by C18:1, which increased from 18.1 ± 3.25% (CONTROL) and 22.1 ± 3.62% (TM) before the first meal to 43.6 ± 1.80% (CONTROL) and 42.7 ± 2.62% (TM) after the third meal (p < 0.001).

Regarding polyunsaturated fatty acids (**PUFAs**), a decrease in n-6 PUFA levels was observed depending on nutritional condition (NC), with values dropping sharply from baseline (34.9 ± 2.28% for CONTROL and 32.4 ± 1.85% for TM at H0-M1) to a minimum at H6-M3 (10.3 ± 2.28 for CONTROL and 9.01 ± 1.49% for TM)(p < 0.001), corresponding to a reduction of 70.5% for CONTROL and 72.2% for TM. After this marked decline in n-6 PUFA levels, a partial recovery was observed at H24-M3, with values rising to 23.7 ± 1.21% for the CONTROL group and 22.8 ± 6.52% for the TM group (p < 0.001). This pattern was primarily driven by C20:4 n-6 (arachidonic acid) and C18:2 n-6 (linoleic acid), which are the most abundant n-6 PUFAs and showed the most pronounced decreases in their relative proportions during the refeeding phases. Similarly, n-3 PUFA levels also showed a significant decrease, dropping from 3.08 ± 0.30% for CONTROL and 2.65 ± 0.204% for TM before the first meal to a minimum of 0.688 ± 0.239% for CONTROL and 0.897 ± 0.522% for TM, 24 hours after the third meal (p < 0.001).

***Thermal Manipulation condition Effect.*** Thermal manipulation also had an impact on hepatic fatty acid composition. First, when focusing on total SFAs, an interaction effect was observed between TM and NC (p < 0.001), with notable differences between the CONTROL and TM groups at specific time points: at H0-M1, values were 40.0 ± 0.88% for the CONTROL group compared to 37.1 ± 0.93% for the TM group, representing a 7.3% decrease. At H6-M1, values were 44.4 ± 1.52% for CONTROL vs. 47.4 ± 1.69% for TM (7.7% higher) (p < 0.001). Thermal treatment also specifically affected the proportions of minor SFAs, with a positive effect on C14:00 and a negative effect on C15:00 (p = 0.049 and 0.004, respectively). An interaction between these two factors resulted in higher proportions of C17:00 in the TM group compared to the CONTROL group at H6-M1 (p < 0.001).

A significant impact of TM was also observed for sum of MUFAs and C16:1 and C18:1 (p < 0.001; p = 0.004 and p < 0.001 respectively), notably through a strong interaction effect (p < 0.001 for all parameters). The sum of MUFAs was approximately 47% higher (26.3 ± 1.36% vs. 17.9 ± 1.48%) at H6-M1 in the TM group compared to the CONTROL group, C16:1 approximately 193% higher (1.48 ± 0.554% vs. 0.506 ± 0.168%), and C18:1 approximately 43% higher (24.2 ± 1.31% vs. 16.9 ± 1.45%).

No significant effect of TM was observed on the total n-6 PUFA percentage. However, TM was found to specifically decrease C18:2 n-6 and C22:4 n-6 (p = 0.016 and p < 0.001, respectively), with an interaction effect observed exclusively for C18:2 n-6 and C20:2 n-6 (p < 0.001 and p = 0.002, respectively). Notably, a significant difference between the TM and CONTROL groups was observed at time point H6-M1 for C18:2 n-6, with the TM group exhibiting a mean value 30.3% lower (8.16 ± 0.939%) compared to the CONTROL group (11.7 ± 1.77%).

Regarding the average n-3 PUFA levels, a decrease was observed in TM compared to CONTROL (p = 0.013), as well as an interaction between the two factors (p = 0.006). A decrease in the average level of C16:4 n-3 and C22:6 n-3 was also measurable in TM versus CONTROL group.

### Relative gene expression in livers during the fattening phase in the CONTROL and TM groups

To support the physiological observations, we analyzed the relative expression of genes involved in carbohydrate and lipid metabolism in the livers of adult animals (Table 4).

**Table 4:** Hepatic relative expression of genes involved in lipid metabolism and glucose transport in CONTROL and TM groups.

							Anova two ways or Scheirer-Ray-Hare test			
Nutrionnal Condtion (NC)							NC effect	TM effect	Int	n
		H0-M1	H6-M1	H6-M2	H6-M3	H24-M3	P	P	P	
Gene	Group	23 h Fast	6 h post meal	6 h post meal	6 h post meal	23 h Fast				
Carbohydrate Transport										
*GLUT2*	CONTROL	1.19 ± 0.79^a^	0.42 ± 0.34^a^	2.45 ± 1.63^a^	1.54 ± 0.62^a^	0.74 ± 0.32^a^	0.032	0.94	0.034	6 to 7
	TM	0.69 ± 0.56^a^	3.22 ± 3.56^a^	1.08 ± 0.34^a^	1.63 ± 1.21^a^	1.02 ± 0.62^a^				
Carbohydrate Oxidation										
*ENO1*	CONTROL	1.09 ± 0.58	0.51 ± 0.28	1.39 ± 0.58	1.83 ± 0.75	1.21 ± 0.38	< 0.001	0.75	0.361	6 to 8
	TM	0.84 ± 0.36	0.93 ± 0.34	1.18 ± 0.24	1.92 ± 0.70	1.01 ± 0.19				
*GAPDH*	CONTROL	1.49 ± 0.92	0.891 ± 0.48	1.17 ± 0.38	1.30 ± 0.23	1.58 ± 0.42	0.041	0.86	0.595	6 to 10
	TM	1.48 ± 0.38	1.33 ± 0.68	1.03 ± 0.39	1.29 ± 0.52	1.52 ± 0.19				
Lipid Synthetisis										
*ACLY*	CONTROL	0.29 ± 0.22^b^	0.47 ± 0.25^b^	2.38 ± 0.97^a^	1.42 ± 0.61^a^	0.24 ± 0.08^b^	< 0.001	< 0.001	< 0.001	5 to 9
	TM	0.26 ± 0.11^b^	2.40 ± 0.95^a^	3.51 ± 1.68^a^	2.86 ± 2.51^a^	0.20 ± 0.03^b^				
*ACC*	CONTROL	0.71 ± 0.64^bcd^	0.38 ± 0.17^bce^	1.96 ± 0.99^ad^	1.83 ± 0.60^ade^	0.37 ± 0.11^c^	< 0.001	0.91	0.044	6 to 9
	TM	0.62 ± 0.29^bc^	1.70 ± 1.00^ba^	1.82 ± 0.72^ade^	1.74 ± 1.07^ba^	0.34 ± 0.10^c^				
*FASN*	CONTROL	0.24 ± 0.05^cd^	0.72 ± 0.42^b^	2.47 ± 1.04^a^	1.71 ± 0.76^a^	0.09 ± 0.03^c^	< 0.001	< 0.001	0.009	6 to 9
	TM	0.26 ± 0.18^bd^	3.36 ± 1.04^a^	3.68 ± 1.80^a^	2.81 ± 2.47^a^	0.16 ± 0.07^dc^				
*SCD1*	CONTROL	0.21 ± 0.18^c^	0.21 ± 0.21^c^	4.81 ± 4.48^ba^	4.81 ± 1.76^a^	0.12 ± 0.10^c^	< 0.001	< 0.001	0.007	6 to 9
	TM	0.63 ± 0.39^cb^	4.51 ± 4.05^ba^	3.59 ± 1.68^ba^	5.82 ± 4.86^a^	0.41 ± 0.32^c^				
*ELOVL6*	CONTROL	0.55 ± 0.22^c^	0.54 ± 0.21^c^	1.40 ± 0.29^ba^	2.25 ± 0.99^a^	0.47 ± 0.18^c^	< 0.001	< 0.001	0.002	6 to 9
	TM	0.63 ± 0.20^c^	1.61 ± 0.47^a^	1.87 ± 0.52^a^	2.28 ± 0.97^a^	0.78 ± 0.26^cb^				
Lipid Oxidation										
*ACOX1*	CONTROL	1.97 ± 1.06^bca^	0.65 ± 0.28^e^	0.99 ± 0.34^bcde^	0.77 ± 0.22^de^	2.15 ± 0.61^ab^	< 0.001	0.59	0.002	6 to 9
	TM	1.27 ± 0.90^bcde^	1.66 ± 0.75^bcda^	0.95 ± 0.35^cde^	0.72 ± 0.37^e^	2.96 ± 1.52^a^				
*CPT1A*	CONTROL	4.01 ± 1.87^ab^	0.40 ± 0.14^d^	0.52 ± 0.15^cd^	2.25 ± 1.25^be^	5.18 ± 1.23^a^	< 0.001	0.01	< 0.001	6 to 9
	TM	2.31 ± 0.76^b^	0.89 ± 0.2^c^	0.44 ± 0.18^d^	0.96 ± 0.37^ce^	5.88 ± 1.78^a^				
*ACLSL1*	CONTROL	2.30 ± 1.29	0.48 ± 0.11	1.07 ± 0.53	1.55 ± 1.30	3.54 ± 1.64	< 0.001	0.66	0.097	6 to 7
	TM	2.06 ± 0.46	1.07 ± 0.62	0.56 ± 0.17	1.59 ± 1.03	4.59 ± 2.53				
*ACAD11*	CONTROL	1.6 ± 0.92^ab^	1.42 ± 0.90^ab^	1.27 ± 0.76^ab^	0.69 ± 0.44^b^	1.18 ± 0.66^ab^	0.014	0.76	0.05	6 to 9
	TM	1.26 ± 0.38^ab^	1.02 ± 0.39^ab^	1.25 ± 0.63^ab^	0.87 ± 0.33^b^	2.18 ± 0.93^a^				
*ACAT1*	CONTROL	2.20 ± 1.56	1.58 ± 0.80	0.60 ± 0.15	0.76 ± 0.31	4.1 ± 1.46	< 0.001	0.03	0.694	6 to 8
	TM	1.12 ± 0.78	1.33 ± 0.90	0.54 ± 0.16	0.65 ± 0.28	3.53 ± 1.86				
Lipid Transport										
*APOB*	CONTROL	1.05 ± 0.64	1.02 ± 0.33	1.48 ± 0.49	1.02 ± 0.55	0.78 ± 0.32	< 0.001	0.5	0.112	6 to 9
	TM	0.68 ± 0.43	1.83 ± 0.88	1.50 ± 0.32	0.97 ± 0.32	0.82 ± 0.36				
*MTTP*	CONTROL	1.02 ± 0.65^abc^	0.52 ± 0.26^b^	1.27 ± 0.31^abc^	1.74 ± 0.65^c^	1.14 ± 0.30^abc^	0.001	0.3	0.034	6 to 9
	TM	0.68 ± 0.42^ab^	1.55 ± 0.67^abc^	1.48 ± 0.20^abc^	1.89 ± 0.96^ac^	1.04 ± 0.33^abc^				
*LDLR*	CONTROL	1.83 ± 1.23^a^	0.56 ± 0.22^b^	1.30 ± 0.51^ab^	1.31 ± 0.41^ab^	0.84 ± 0.22^ab^	0.191	0.27	0.004	6 to 8
	TM	0.60 ± 0.39^b^	1.26 ± 0.68^ab^	1.18 ± 0.33^ab^	1.08 ± 0.70^ab^	0.81 ± 0.40^ab^				

Values are presented as mean ± standard deviation (SD) for each experimental group (H0-M1, H6-M1, H6-M2, H6-M3, H24-M3), with CONTROL and TM groups. Genes analyzed include those involved in glucose oxidation and transport (*ENO1, GAPDH, GLUT2*), lipid biosynthesis (*ACLY, ACC, FASN, SCD1, ELOVL6, ACAT1*), lipid oxidation (*ACOX, CPT1A, ACSL1, ACAD11*), lipid transport (*APOB, MTTP, LDLR*). Statistical analyses were performed using generalized least squares (GLS) for *ACC* and *GLUT2*; non-parametric Scheirer-Ray-Hare tests followed by Dunn’s post-hoc test for MTTP; two-factor ANOVA on logarithmically transformed data followed by Tukey’s post-hoc test for *FASN, SCD1, ELOVL6, ACLY, ACAT1, ACOX, CPT1A, ACSL1*, and *APOB*; and two-factor ANOVA on raw data followed by Tukey’s post-hoc test for *ENO1, GAPDH, ACAD11*, and LDLR. The effects tested were NC (Nutritional Condition), TM (Thermal Manipulation), and Int (Interaction). Statistical significance is indicated as: ***p < 0.001, **p < 0.01, *p < 0.05, ns = not significant (p ≥ 0.05).

***Nutritional Condition Effect****:* Concerning genes involved in glucose transport and oxidation, an NC effect was measured with increased expression after meals for *GLUT2* (p = 0.009) and *ENO1* (p < 0.001), before returning to baseline values after 24 hours of fasting. The relative expression of *GAPDH* was only slightly decreased on average by meals compared to fasting conditions (p = 0.041).

At the same time, the relative expression of *ACLY, ACC*, and *FASN* involved in lipid biosynthesis exhibited a progressive increase, peaking 6 hours after the second fattening meal (H6-M2) for both groups, with expression levels significantly higher than at H0-M1 (p < 0.001, p = 0.001, and p < 0.001, respectively). After this peak, their expression gradually returned to baseline levels 24 hours after the third meal (H24-M3). In contrast, *SCD1* and *ELOVL6* followed a similar trend but reached their peak expression later, at H6-M3, with significantly higher levels compared to H0-M1 (*p* < 0.001 for both genes).

We then focused on genes involved in lipid oxidation, with the average relative expression of *ACOX, ACSL1, CPT1, ACAT1, and ACAD11* significantly decreased after meals compared to fasting conditions (NC effect, p < 0.001 for the first four genes and p = 0.014 for *ACAD11*). Expression levels then increased after fasting, reaching their peak 24 hours after the third meal (H24-M3).

Finally, regarding the expression of genes involved in lipid transport, a significant increase was observed between H0-M1 and H6-M2 for *APOB* (*p* = 0.002) and between H0-M1 and H6-M3 for *MTTP* (*p* = 0.001) for the CONTROL and TM groups. Both genes returned to their initial levels at H24-M3.

***Thermal Manipulation Effect.*** Regarding carbohydrate metabolism, only the *GLUT2* transporter was affected by TM, with a significant interaction effect with NC (p = 0.034). However, no differences were detected in the post-hoc analyses.

Among genes involved in lipid biosynthesis, TM significantly affected the expression of all genes except *ACC* (p < 0.001). Moreover, an interaction between TM and nutritional condition (NC) was observed for all genes, revealing at H6-M1 a higher relative expression levels in the TM group for *ACLY, FASN, SCD1* and *ELOVL6*. For genes related to lipid oxidation, interaction effects between NC and TM were also observed for the genes *ACOX, CPT1, ACAD11*, and *ALDH7A1*. Notably, at H6-M1, the TM group showed higher expression levels compared to the CONTROL group for *ACOX, CPT1*, (p = 0.011 and p = 0.025 respectively). Regarding *ACAT1* relative expression, a global effect of TM was observed, with overall lower expression levels in the TM group compared to the CONTROL group (p = 0.025). Finally, regarding lipid transport, an interaction effect was observed with the NC effect for the genes *MTTP* (p = 0.034) and *LDLR* (p = 0.004). Notably, a significant difference was detected at H0-M1, with the CONTROL group showing higher expression levels of *LDLR* compared to TM group (p = 0.021).

## Discussion

This study focused on short-term hepatic responses and aimed to investigate whether hepatic fattening in ducks can be enhanced by combining two factors: a fractionated feeding regimen designed to induce voluntary hyperphagia, and embryonic thermal manipulation, which is expected to trigger lasting metabolic adaptations favoring energy storage in the form of lipids. To address these objectives, the effects of each factor were evaluated individually, as well as their combined effect, on various zootechnical and hepatic parameters.

### The thermal stimulus was perceived at the embryonic level

We first examined the effects of TM on the relative expression of target genes in the liver of embryos at the end of the programming period (day 21 of incubation). As shown in Table 1, the TM group exhibited increased expression of *HSP90, HSP10, HSF5*, and *UBQLN1*, genes involved in cellular stress responses (Velichko et al., 2013; Balakrishnan et al., 2023; Phung et al., 2023; Gouda et al., 2024), and up-regulated in response to a thermal changes in different species (Vinoth et al., 2015; Massimino et al., 2021; Ncho et al., 2022; Satapathy et al., 2023). These results therefore suggest that thermal stimulus was indeed experienced by the embryos of the TM group. An increased relative expression was also observed for *DIO3* and *ALB*, both involved in the thyroid signaling pathway (Lisowska-Myjak et al., 2021) as well as *TYMS* and *ELP3*, which play key roles in epigenetic processes (Winkler et al., 2002; Hernandez and Stohn, 2018). These upregulations confirm that these pathways are cellular targets of TM (Yiangou et al., 1998; Al-Zghoul et al., 2015; Massimino et al., 2021; Andrieux et al., 2025).

Embryonic TM resulted in increased late embryonic mortality and reduced hatchability compared with the CONTROL group, in contrast with our previous findings (Andrieux et al., 2023b). However, substantial inter-experimental variability has already been reported under similar conditions (Andrieux et al., 2022), highlighting the sensitivity of this approach to experimental context. Overall, these results suggest that hatchability outcomes are highly dependent on incubation conditions, emphasizing the need for further refinement of the thermal protocol. Besides, the reduction in hatchability mainly affected females, resulting in a male-biased sex ratio in the TM group, which suggest a greater sensitivity of female embryos to TM, as previoulsy reported in duck (Andrieux et al., 2022), or chicken (Tzschentke and Halle, 2009, Elmehdawi et al., 2015). Overall, these results suggest that the thermal level applied in the present study may be close to or above the optimal range for embryonic thermal programming, and that the current TM conditions are not yet fully optimised, particularly regarding embryonic survival and sex-dependent sensitivity, and would benefit from further refinement.

For subsequent analyses, only males, whose survival was not affected by the TM, were retained to avoid a bias that could have arisen from the selective loss of the most fragile females, potentially reflecting a selection effect rather than true programming. In addition, this choice is consistent with standard industrial practices, as females are not typically used in conventional *foie gras* production protocols (Atallah et al., 2024).

### The third fattening meal, following the acclimation phase, induced a transient increase in hepatic lipid storage

To address the first objective of this study, which aimed to assess the short-term effect of three fattening meals administered at 12-hour intervals on hepatic lipid storage, we focused here exclusively on the effects of the nutritional condition (NC).

First, we focused on the impact of fractionated feeding (NC effect) on feed intake, which corresponds to the pen-level standardized intake (per bird equivalent). The increase in feed intake observed with 24 h meal intervals suggests a progressive adaptation of feeding behaviour to the fractionated feeding schedule, consistent with previous reports indicating that a short acclimation period may be required to induce hyperphagia in mule ducks (Zwick et al., 2026). These results are particularly consistent with studies in birds, which have shown that feeding at fixed times promotes the development of anticipatory behaviors and adaptations in food intake (Benedict et al., 2023; Parker et al., 2025). During the fattening phase (12 h meal intervals), feed intake remained stable across the first two meals, reaching an average of 646 g per 24 h. This level is markedly higher than *ad libitum* intake in Pekin and mule ducks (∼250 g/day)(Bley and Bessei, 2008; Basso et al., 2014) and falls within the range observed under conventional force-feeding protocols (400-800 g/day)(Bonnefont et al., 2019). These results provide evidence that the ducks in this study developed hyperphagic behavior, although this response appeared transient, with feed intake significantly decreasing as early as the third fattening meal (M3, 12 h apart). This highlights both the limitations of the protocol and the rapid adaptive capacity of mule ducks in regulating feed intake (Knudsen et al., 2017). One hypothesis is that, when the feeding regimen was altered to three meals spaced 12 hours apart, the animals initially exhibited hyperphagic behavior due to a temporarily unpredictable perception of meal timing. By the third meal (M3), the schedule may have become predictable again, leading to reduced intake. This is consistent with evidence in birds that feeding predictability influences intake and behaviour (Boon et al., 1999; Lynn et al., 2023; Parker et al., 2025).

The increase in body weight after meals compared with fasting periods is mainly due to feed intake during the first two meals of the fattening phase, increasing body weight through both digestive contents and energy storage in multiple tissues, including the liver. Conversely, a decrease in body weight during fasting, as observed in chickens, results from energy mobilization and gastrointestinal emptying (Xue et al., 2021). Plasma glucose was lowest at H24-M3, likely reflecting the low intake at M3 (90 g) and the subsequent 23 h fast, while remaining stable at other time points. In force-fed ducks, glycaemia typically peaks around 1 h post-meal (Tavernier et al., 2017). Sampling after a long fast or six hours post-feeding may explain the lack of significant differences. With regard to TG, lower concentrations were observed in fasting conditions compared to measurements taken six hours after meals, which is in line with the metabolic responses expected in this type of protocol carried out in avian species (Anthony et al., 1990; Chen et al., 2021; Liu et al., 2024). In contrast, concentrations of NEFAs were higher during fasting and decreased after refeeding, reflecting increased mobilization of lipid reserves during fasting and reduced postprandial lipolysis (Anthony et al., 1990; Zhou et al., 2005; Serr et al., 2009; Wang et al., 2017; Xiao et al., 2020). These results confirm that the animals consumed the feed provided during refeeding phases, as reflected by expected metabolic differences between fasting and post-meal periods. Despite the low intake at M3, plasma TG and FFA levels still indicated a postprandial state after 6 h, which may reflect either a sufficient metabolic response to a small meal or a delayed lipid metabolism influenced by the preceding higher-intake meals.

Liver weight, hepatic glycogen, total lipids, and TG were then assessed. Liver weight and glycogen increased up to 6 h after the second fattening meal, whereas lipid accumulation remained limited, consistent with previous studies (Andrieux et al., 2023a; Zwick et al., 2026). In contrast, the third meal induced an increase in hepatic lipids and TG, accompagnied by a decrease in glycogen, while liver weight remained stable despite reduced intake. This suggests a shift in hepatic energy partitioning, as previously described during force-feeding (Bonnefont et al., 2019), or may also reflect glycogen mobilization in response to reduced feed intake. In this protocol, liver weight is approximately 150 g, with a total lipid content approaching 10%, and a glycogen content representing 7% of liver weight. By comparison, after three force-feeding meals, 12-week-old animals displayed liver weighing 200 g with a lipid content of about 15% and a glycogen level approaching 10% (Bonnefont et al., 2019). These differences could be explained by the quantity of feed ingested during the third meal, the nature of the feed (60% starch for overfeeding meals versus 40% in the growth feed used in the study), and the age of the animals. Furthermore, in this study, a 24 h fast reversed these effects, with liver weight, TG and total lipids returning to baseline and glycogen reserves being almost completely depleted. This suggests that the protocol induces a transient lipid deposition. It also supports the notion that hepatic storage is a highly reversible process, as observed following the cessation of force-feeding, where hepatic lipid and glycogen stores are progressively mobilized and hepatic parameters return to baseline levels within approximately 29 days (Bernardi et al., 2026).

Hepatic fatty acid composition was strongly influenced by the fractionated feeding protocol. Palmitate (C16:0) showed a moderate postprandial increase, while stearic acid (C18:0) decreased after meals and increased during fasting, in contrast to MUFAs, mainly oleic acid (C18:1), which increased markedly. This reciprocal pattern suggests enhanced SFA desaturation, likely involving stearoyl-CoA desaturase 1 (SCD1), and is consistent with previous findings under fasting-refeeding and force-feeding conditions (Molee et al., 2005; Chartrin et al., 2006; Andrieux et al., 2023a). In parallel, PUFA levels decreased postprandially, in agreement with previous observations in both fasting-refeeding and force-feeding contexts (Molee et al., 2005; Chartrin et al., 2006; Andrieux et al., 2023a). Twenty-four hours after the third fattening meal, a partial return toward baseline levels was observed. However, MUFA proportions remained elevated compared to pre-feeding levels, suggesting that hepatic lipid reserves persist longer than glycogen, which was nearly depleted at this stage of the protocol.

The physiological changes observed are supported by molecular data showing clear postprandial regulation of key metabolic genes. Refeeding phases induced upregulation of genes involved in glucose transport/oxidation (*GLUT2, ENO1*) and lipid synthesis (*ACLY, ACC, FASN, SCD1, ELOVL6*), whereas fasting resulted in their downregulation. These patterns are consistent with previous observations in ducks and other avian species under both voluntary feeding and force-feeding conditions (Serr et al., 2009; Fang et al., 2014; Tavernier et al., 2018; Cogburn et al., 2020; Chen et al., 2021; Massimino et al., 2021; Andrieux et al., 2023a). Conversely, genes involved in lipid oxidation (ACOX, CPT1, ACSL1, ACAD11, ACAT1) were downregulated, showing an expression pattern opposite to that of lipid synthesis genes, in agreement with previous findings in chickens, geese, and ducks (Fang et al., 2014; Tavernier et al., 2018; Chen et al., 2021; Massimino et al., 2021; Andrieux et al., 2023a). These results indicate that fractionated feeding promotes the transcriptional activation of lipid synthesis from dietary glucose. However confirming the involvment of *de novo* lipogenesis in this process will require additional analyses at the protein level and of enzymatic activity. Interestingly, expression remained elevated after the third fattening meal despite reduced intake, indicating a sustained transcriptional response partly independent of immediate feed consumption, in line with previous findings (Zwick et al., 2026).

### Thermal manipulation induced earlier molecular changes, enhancing hepatic lipid storage

The second objective of our study was to determine whether applying TM during embryogenesis could optimize hepatic metabolism within the context of a fractionated feeding protocol.

Regarding the impact of TM, no significant effects were observed on feed intake or on circulating FFA and plasma glucose. However, body weight was globally affected by TM, independently of nutritional condition. Similar findings have previously shown that TM can positively influence body weight (Ncho et al., 2021). Plasma TG levels were also positively affected by TM. Concerning liver energy composition, TM positively influenced liver weight, as well as hepatic TG and total lipid content, whereas hepatic glycogen remained unaffected. All parameters affected by TM followed a similar pattern, with higher levels in the TM group after the first fattening meal. This effect was transient, as values became comparable between groups from the second meal onwards. Plasma TG, as well as hepatic TG and total lipid contents, were increased in the TM group after the first meal, indicating enhanced lipid synthesis and likely explaining the higher liver weight.

Concerning hepatic fatty acid composition, previous studies have shown that embryonic TM can modify fatty acid proportions in mule ducks, both under force-feeding (Andrieux et al., 2023b) and fasting-refeeding conditions (Andrieux et al., 2023a). In our study, TM also altered the relative distribution of SFA, MUFA, and PUFA within hepatic lipids. After the first meal, higher SFA and MUFA levels, particularly oleate (C18:1), were observed in the TM group, whereas PUFA showed the opposite trend, suggesting an earlier activation of lipogenesis. These changes are consistent with enhanced hepatic lipid synthesis, as oberved under force-feeding conditions (Molee et al., 2005; Chartrin et al., 2006).

The differences observed in hepatic fatty acid composition of the TM group can be explained by transcriptional regulation of lipid metabolism. The expression of key lipogenic genes (*FASN, SCD1*, and *ELOVL6*) was strongly affected by TM, with an early induction already detectable after the first fattening meal. *FASN* is involved in palmitate synthesis from malonyl-CoA, while *ELOVL6* in elongation of fatty acids from C16:0 to C18:0, and *SCD1* in conversion of C18:0 into oleic acid (C18:1)(Collins et al., 2011). These changes are consistent with previous studies showing that embryonic TM upregulates hepatic lipogenesis in both chickens and ducks (Loyau et al., 2014; Massimino et al., 2021; Andrieux et al., 2023a; Li et al., 2024), and may explain the higher C18:1 levels observed in the TM group after the first meal. Expression levels in the CONTROL group subsequently reached those of the TM group after the second meal, indicating a transient effect of TM on lipogenesis. In parallel, genes involved in lipid oxidation (*ACOX, CPT1)* were also upregulated after the first meal in the TM group, suggesting that TM affects both lipid synthesis and catabolism at the molecular level. In a previous study conducted in ducks, similar patterns were observed during embryonic thermal programming, with an increase in the expression of pathways involved in lipid oxidation alongside those involved in lipid storage (Andrieux et al., 2023b). In the current context, the increased expression of lipid oxidation genes in the TM group may represent a compensatory response to higher lipid availability rather than a direct increase in oxidative capacity. However, despite this enhanced lipid oxidation, overall lipid storage remained greater in the TM group, as reflected by the higher total hepatic lipid content observed after the first fattening meal. Thus, TM appears to induce a coordinated reorganization of hepatic lipid metabolism, affecting both synthesis and oxidation while favoring net lipid accumulation in the liver.

In summary, these results suggest that embryonic TM induces a temporally regulated reprogramming of hepatic lipid metabolism, characterized by an early and coordinated activation of both lipid synthesis and oxidation pathways during the first meal, followed by a progressive attenuation of these differences over subsequent meals.

## Conclusion

The fractional feeding protocol led to the development of a hyperphagic behavior, which, however, proved to be transient, with a marked decrease in feed intake at the third fattening meal. It also resulted in a significant increase in liver weight, associated with higher glycogen, total lipid, and triglyceride contents, as well as increased oleate synthesis. This effect was also transient, as parameters returned to baseline levels after a 24-hour after the last meal. Regarding TM, although it did not lead to increased liver weight or lipid content at the end of the protocol, it induced a temporal shift in energy storage mechanisms, characterized by an earlier activation of these processes in the TM group. Although this protocol remains far from providing a viable alternative to force-feeding ducks for long-term *foie gras* production, it could serve as a foundation for developing novel alternative strategies.

## Aknowledgments

We would like to thank the “Comité Interprofessionnel des Palmipèdes à Foie Gras” (CIFOG), the “Conseil Général des Landes” (CD40) and the PHASE department of INRAE for their financial support. Thanks also to the staff and technicians of the waterfowls experimental station (UEPFG, INRAE) for taking care of the animals during the two experiments and for their advice.

## CRediT authorship contribution statement

**Laura-Lou Zwick:** Writing – review & editing, Writing – original draft, Visualization, Validation, Methodology, Formal analysis, Data curation, Conceptualization. **Joséphine Huot:** Writing – original draft, Formal analysis, Data curation. **Cécile Heraud:** Writing – original draft, Formal analysis, Data curation. **Marie Lasserre:** Writing – original draft, Data curation. **Anne Surget:** Writing – original draft, Formal analysis, Data curation. **Karine Gontier:** Writing – original draft, Validation, Supervision, Data curation. **Jérôme Roy:** Writing – review & editing, Writing – original draft, Validation, Supervision, Methodology, Conceptualization. **Stéphane Panserat:** Writing – review & editing, Writing – original draft, Visualization, Validation, Supervision, Formal analysis, Conceptualization. **Marianne Houssier:** Writing – review & editing, Writing – original draft, Visualization, Validation, Supervision, Project administration, Methodology, Investigation, Funding acquisition, Formal analysis, Conceptualization.

## Disclosures

The authors declare the following financial interests/personal relationships which may be considered as potential competing interests: HOUSSIER Marianne reports financial support was provided by Comité Interprofessionnel des Palmipèdes à Foie Gras (CIFOG). ZWICK Laura-Lou reports financial support was provided by Comité Départemental des Landes (CD40). ZWICK Laura-Lou reports financial support was provided by INRAE département PHASE. If there are other authors, they declare that they have no known competing financial interests or personal relationships that could have appeared to influence the work reported in this paper.

## Appendix Supplementary materials

Image, application 1

Image, application 2
