# Supplementary material for: Embryonic thermal programming combined with fractionated feeding induces early activation of hepatic lipid storage pathways in mule ducks
Source: Poult Sci. 2026 May 7;105(9):107075. doi: 10.1016/j.psj.2026.107075 (PMC13226918; doi:10.1016/j.psj.2026.107075)
Supplement: Supplementary file 1 [file mmc1.docx]

**Supplemental Table 1.** Detailed Composition of growth feed (PAG1320, Nutricia, route de Saint Sever, Haut Mauco, BP27, France).

| Commercial Diet PAG 1320 | | |
| --- | --- | --- |
| Category | Constituent / Additive | Quantity |
| Analytical Constituents | Dry Matter (DM) | 87,18% |
|  | Crude Protein | 16.45% DM |
|  | Crude Fat | 2.25% DM |
|  | Crude Ash | 4.57 % DM |
|  | Starch | 48.52 % DM |
|  | Energy (kJ.g−1) | 18,45 |
| Essential amino acids and minerals | Lysine | 0.69 % |
|  | Methionine | 0.29 % |
|  | Calcium | 0.95 % |
|  | Phosphorus | 0.45 % |
|  | Sodium | 0.15 % |
| Vitamins | Vitamin D3 (3a671) | 3000 IU/KG |
|  | Vitamin A (3a672a) | 7000 IU/KG |
| Amino Acids | Hydroxyl Analog of Methionine | 0.11 % |
| Digestibility Enhancers | Endo-1,4-Beta-xylanase (EC 3.2.1.8) | 150 FXU/KG |
|  | 4a19 6.Phytase (EC 3.1.3.26) | 1000 FTU/KG |
| Trace Elements | Copper (3b4O5 sulfate II pentahydrate) | 15 MG/KG |
|  | Iron (3b103 sulfate (II) monohydrate) | 24 MG/KG |
|  | Manganese (3b502 oxide) | 50 MG/KG |
|  | Iodine (3b201 potassium iodide) | 1.20 MG/KG |
|  | Zinc (3b605 monohydrate sulfate) | 35 MG/KG |
|  | Selenium (sodium selenite 3b801) | 0.20 MG/KG |
| Main Ingredients | Wheat | - |
|  | Southwest Corn (Class A) | - |
|  | Sunflower meal (feed extraction) | - |
|  | Barley | - |
|  | Wheat Bran | - |
|  | Calcium Carbonate | - |
|  | Sodium Chloride | - |

**Supplemental Table 2. Nucleotide sequences of the PCR primers used to evaluate mRNA relative expressions of by real-time quantitative PCR.**

| gene (name) | gene (symbole) | Forward Primer Sequence | Reverse Primer Sequence |
| --- | --- | --- | --- |
| Heat Shock Protein 90 | *HSP90* | AGACCATTTGGCTGTCAAGC | CAGCTCCTCACAGTTGTCCA |
| Heat Shock Protein 10 | *HSP10* | AGTTCCTTCCCCTGTTTGAT | GCTTGTAGCACTTTCCCTTGA |
| Heat Shock Factor 5 | *HSF5* | TCTTGGGTTTGTTCCAGGAC | GCAGTGCAGCTGTTGGATAA |
| Ubiquilin | *UBQLN1* | GTAAGAATGGTGGCCTTGGA | TCTGAACGAATGGGTTTTCC |
| Deiodinase, Iodothyronine Type III | *DIO3* | GCGAGCTTTCGAGCAAGATG | ATCCCGAAGGAAGAGAGCCT |
| Albumin | *GLB* | CCATGGTAGACAAGTGCTGC | TCCTGGAGCCGTGTCTT |
| Fibroblast Growth Factor Receptor 2 | *FGFRF2* | CGCAGGATGGTCCACTCTAT | CCAGCTGGTAAGTCAGGAT |
| Elongator Acetyltransferase Complex Subunit 3 | *ELP3* | GGAGGTGTTTCCATCGTC | TTGGCTATTCTCTCTGCCT |
| Thymidylate Synthase | *TYMS* | AGCCTGGAGAGTTCATACACAC | TTTGAGTTTGGGGAAAGGTC |
| Solute Carrier Family 2 Member 2 | *GLUT2* | GGAGTTGACCZZCCCGTTTA | CCCACCTCGAAGAAGATGAC |
| Enolase 1 | *ENO1* | CGCTACATGGGGAAAGGTGT | AAGGTCAGCAATGTGACGGT |
| Glyceraldehyde-3-Phosphate Dehydrogenase | *GAPDH* | CAGAGGACCAGGTTGTCTCC | CACCACACGGTTGCTGTATC |
| ATP Citrate Lyase | *ACLY* | ACCCCACTGTTGGACTATGC | GCTTCAAGCGCTTCTGATCT |
| Acetyl-CoA Carboxylase | *ACC* | TGCCTCCGAGAACCCTAA | AAGACCACTGCCACTCCA |
| Fatty Acid Synthase | *FASN* | TGAAGAAGGTCTGGGTGGAG | CTCCAATAAGGTGCGGTGAT |
| Stearoyl-CoA Desaturase | *SCD1* | AGTGCTGCTCACATGTTTGG | TGAAGTCGATGAAGGCTGTG |
| ELOVL Fatty Acid Elongase 6 | *ELOVL6* | CTACTTTGTGCTCTTCTGCCATTTT | TGGCTTCTGTCTCATTTCTACCAC |
| Acyl-CoA Oxidase 1 | *ACOX1* | CATGTTTGAGTGGGAAAGA | TTTTCAGGGCAGGAAAATTG |
| carnitine palmitoyltransferase 1A | *CPT1A* | GATTTGGACCAGTGGCTGAT | GAAGGTTGCTTTGCACCAAT |
| Acyl-CoA Synthetase Long Chain Family Member 1 | *ACSL1* | GGCTGGCTTCATACAGGAGA | CTCTTTTCTTGGCCCATTTG |
| Acyl-CoA Dehydrogenase Family Member 11 | *ACAD11* | TGGTTGTACCTCGAGCTGTG | CATCCACATGAGAGGGCTTT |
| Acetyl-CoA Acetyltransferase 1 | *ACAT1* | GGTATCCCCGTGGAAGAAGT | TTCCACCAGCAACCATTACA |
| Apolipoprotein B | *APOB* | TCTCACCGTGACTTGAGTGC | TCCCAGCAGAAGGTGAAGAT |
| Low-Density Lipoprotein Receptor | *LDLR* | TGTGGCCTTCAGAAAGCTCG | ATCTCGTGCTGCATGTAGGG |
| Microsomal Triglyceride Transfer Protein | *MTTP* | TGCAGATGGACAGAGTCGAG | GGATGCAGTGCTGAAAACCT |
| Elongation Factor 1 | *EF1* | AGGTGTCGAGCGTCTTTCTG | TGTAGATGAGGTGGCCGGTA |
| Actin beta | *ACTINB* | CCAGCCATCTTTCTTGGGTA | ATGCCTGGGTACATTGTGGT |
| Luciferase | *LUCIFERASE* | CATTCTTCGCCAAAAGCACTCTG | AGCCCATATCCTTGTCGTATCCC |
