# Supplementary material for: Embryonic thermal programming combined with fractionated feeding induces early activation of hepatic lipid storage pathways in mule ducks
Source: Poult Sci. 2026 May 7;105(9):107075. doi: 10.1016/j.psj.2026.107075 (PMC13226918; doi:10.1016/j.psj.2026.107075)
Supplement: Supplementary file 2 [file mmc2.docx]

**Supplemental Material**

**Additional file 1: Supplementary Tables.** Composition of the starter and growing diets, and list of primers used for RT-qPCR analyses, including sequences and amplicon sizes.
